# Supplementary figures and images for: Comparison of Bioluminescent Substrates in Natural Infection Models of Neglected Parasitic Diseases
Source: Int J Mol Sci. 2022 Dec 16;23(24):16074. doi: 10.3390/ijms232416074 (PMC9781651; doi:10.3390/ijms232416074)

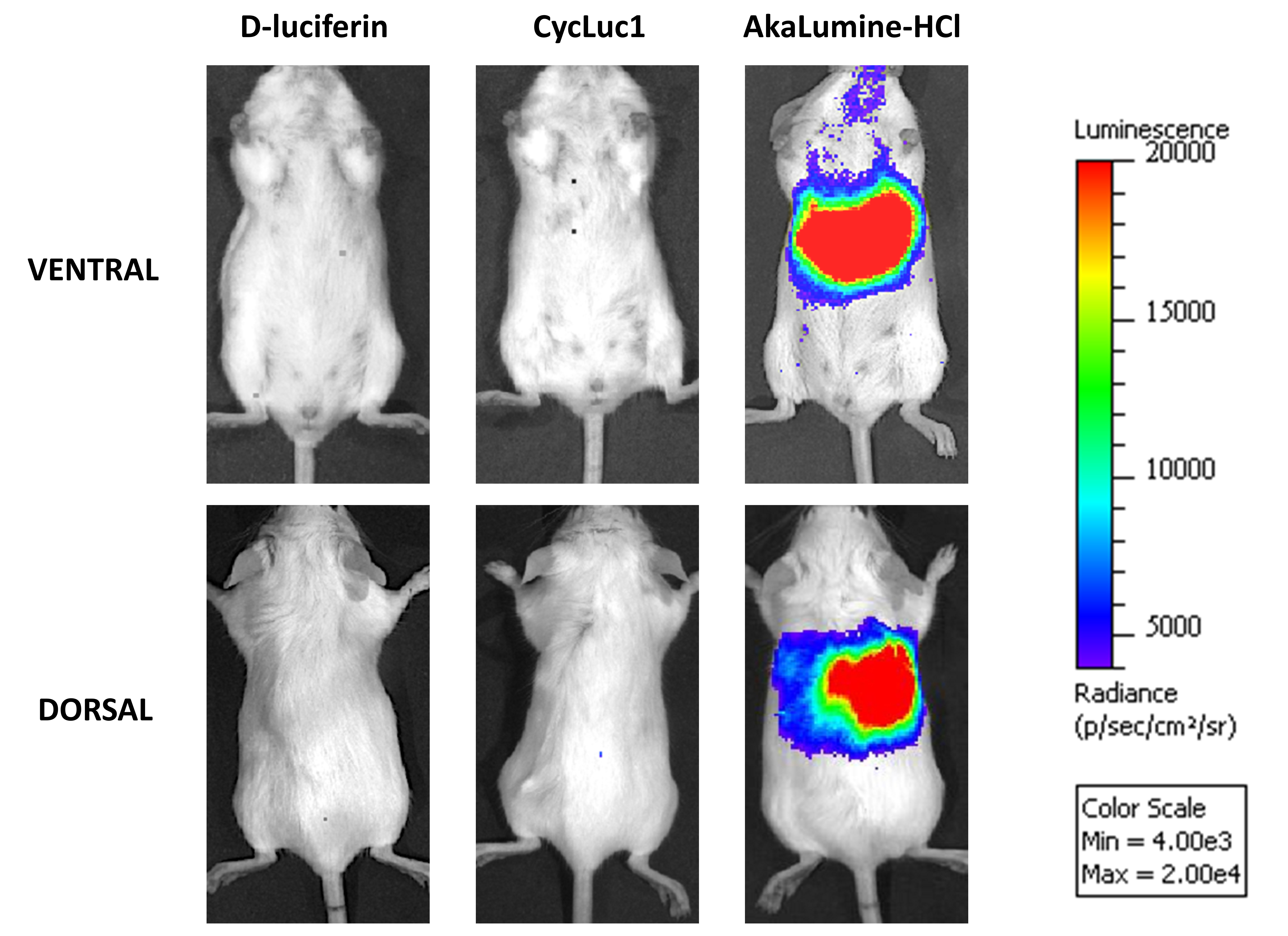

Supplement: Supplementary file 1 [file ijms-23-16074-s001.zip › Figure S1.tif]

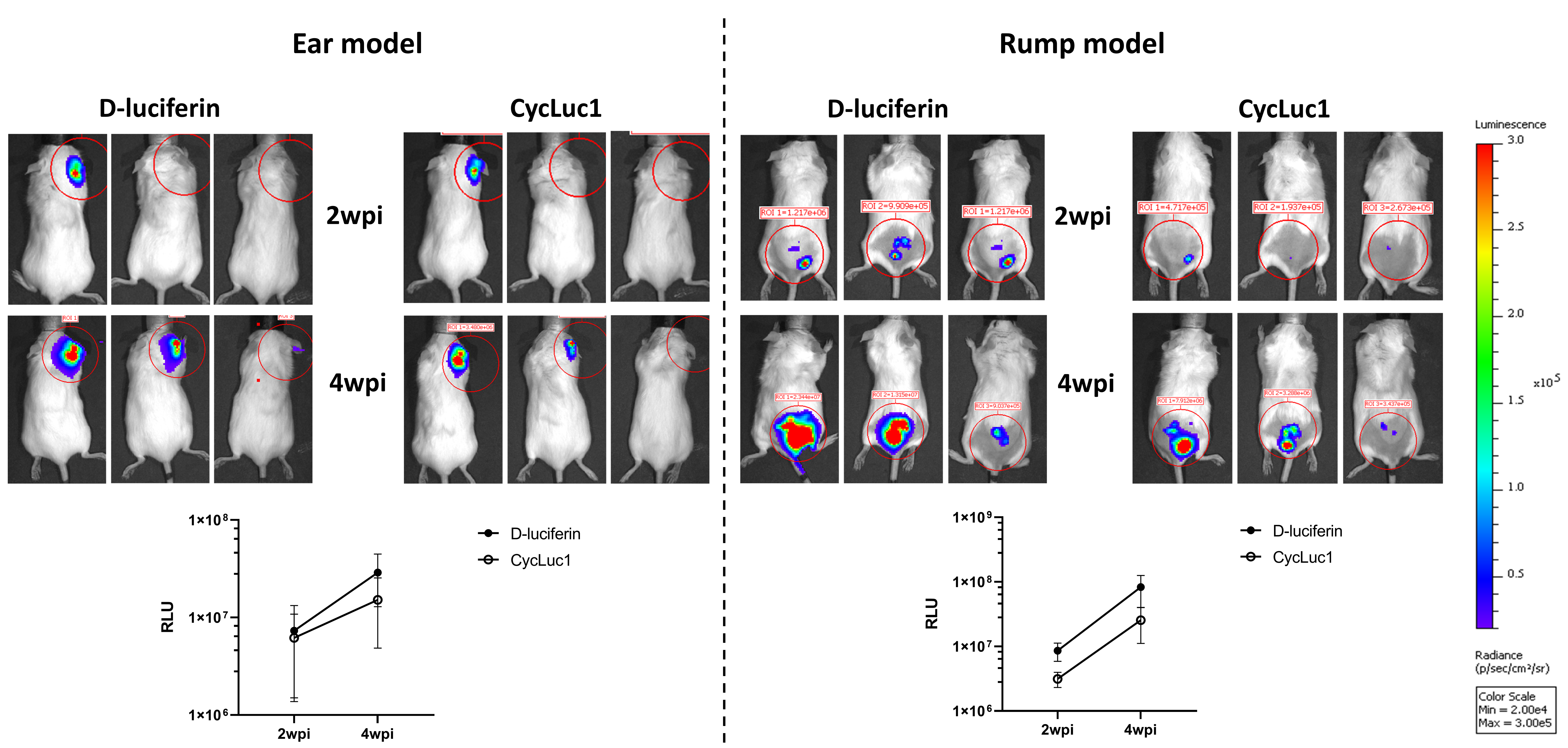

Supplement: Supplementary file 1 [file ijms-23-16074-s001.zip › Figure S2.tif]

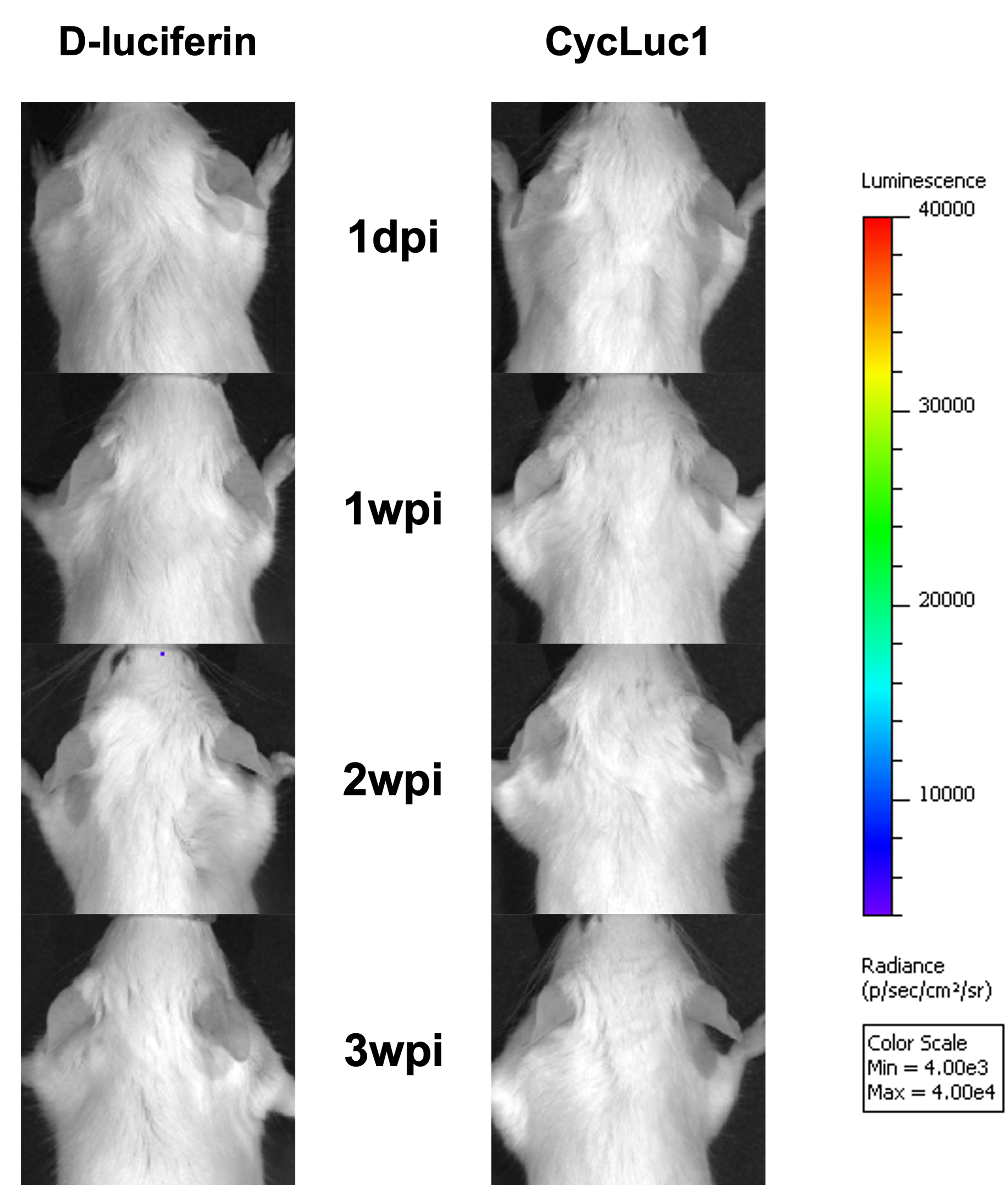

Supplement: Supplementary file 1 [file ijms-23-16074-s001.zip › Figure S3.tif]
